# Spray-Dried Polymeric Microspheres for Lipophilic Drugs: Formulation Design, Physicochemical Characterization, and In Vitro Release Evaluation

**DOI:** 10.3390/ph18071020

**Published:** 2025-07-09

**Authors:** Felipe Nataren-Rodríguez, Jorge Pacheco-Molina, Sandra Leticia Gracia-Vásquez, Isaías Balderas-Rentería, Mónica A. Ramírez-Cabrera, Eder Arredondo-Espinoza, Karla J. Santamaría, Patricia González-Barranco

**Affiliations:** 1 Facultad de Ciencias Químicas, Universidad Autónoma de Nuevo León, Av Universidad S/N, Cd. Universitaria, San Nicolas de los Garza 66455, Nuevo León, Mexico; fnatarenr@uanl.edu.mx (F.N.-R.); sandra.graciavs@uanl.edu.mx (S.L.G.-V.); karla.santamarialpz@uanl.edu.mx (K.J.S.); 2 Facultad de Farmacia, Universidad de Costa Rica, Sede Rodrigo Facio, Montes de Oca, San Pedro 11501-2060, San José, Costa Rica; jorgepac@gmail.com; 3 Laboratorio de Farmacología Molecular y Modelos Biológicos, Facultad de Ciencias Químicas, Universidad Autónoma de Nuevo León—UANL, Av. Guerrero s/n, Col. Treviño, Monterrey 64570, Nuevo León, Mexico; isaias.balderasrn@uanl.edu.mx (I.B.-R.); monica.ramirezcbr@uanl.edu.mx (M.A.R.-C.); eder.arredondosp@uanl.edu.mx (E.A.-E.)

**Keywords:** Eudragit, guar gum, hydroxypropyl methylcellulose, inulin, lipophilic drugs, microsphere

## Abstract

**Background/Objectives**: The formulation of microspheres for lipophilic drugs using aqueous methods, such as spray drying, faces significant challenges. The main objective of this study was to evaluate the effect of the process parameters and polymer selection on the production of microspheres by spray drying for a lipophilic drug. **Methods**: Lipophilic drug-loaded microspheres were developed using various polymers via the aqueous spray drying method. The effects of the factors on the yield percentage and encapsulation efficiency were analyzed. Microspheres preparation included *Agave* inulin, guar gum, hydroxypropyl methylcellulose, and Eudragit^®^ S100. A 2^3^ factorial design was performed, and the parameters were optimized. **Results**: Inlet temperature, feed flow, and polymer percentage showed a significant effect (*p* < 0.05) on the yield percentage of guar gum microspheres and encapsulation efficiency of the inulin microspheres. Inulin and guar gum microspheres showed the best yield percentage (75.41%) and encapsulation efficiency (100%), respectively. In addition, guar gum microspheres had the best morphology, and hydroxypropyl methylcellulose microspheres were smaller and had an irregular surface. Eudragit did not maintain its delayed release property due to limitations of the aqueous method; inulin released the drug immediately, and guar gum and hydroxypropyl methylcellulose microspheres prolonged release only by a few additional hours. **Conclusions**: The experimental design showed that optimizing the parameters (inlet temperature, feed flow, and the type and percentage of polymer) can regulate the microsphere development process to obtain improved product yield and encapsulation efficiency results.

## 1. Introduction

Currently, recent studies are increasingly focused on the development of novel drug delivery strategies to enhance drug bioavailability and mitigate adverse effects. Among these strategies, microcapsules and microspheres have emerged. In microcapsules, the drug is enclosed within a core surrounded by a distinct capsular wall, which is commonly employed to protect the active ingredient and to delay the release of its contents. In contrast, microspheres are pharmaceutical dosage forms where the drug is uniformly dispersed throughout the spherical matrix; they are small spherical particles with diameters ranging from 1 to 100 µm, crafted from both natural and synthetic materials [1,2,3]. They offer the potential for prolonged, delayed, and localized therapeutic effects, thereby reducing dosing frequency and improving patient adherence to therapies. Furthermore, they have the capacity to enhance drug bioavailability and minimize the occurrence or severity of adverse effects [2,4,5].

Today, microsphere production is carried out using a variety of advanced methods, such as emulsification, coacervation, complexation, and nanoprecipitation. The choice of the appropriate method depends entirely on the properties of the polymeric material and the type of drug to be encapsulated [2,6].

For example, the emulsification method, commonly used to encapsulate poorly water-soluble drugs, produces discrete drops or capsules after removing the organic solvent. In contrast, the coacervation technique is applied to encapsulate hydrophobic drugs, leveraging the electrostatic interaction between colloids to achieve spontaneous liquid–liquid phase separation [2,3].

Nanoprecipitation, also known as solvent displacement, is another method used. It is like emulsification but aimed at forming nanoparticles suspended in water after the solvent evaporates. Finally, complexation involves adding active agents to an aqueous solution, followed by the application of heat to obtain the desired complexes [2,6].

Although these methods offer a wide range of possibilities for encapsulating compounds, it is important to note that each has potential disadvantages. These include high cost, significant solvent consumption, and complexity in industrial scale-up. Additionally, the removal of organic solvents used in many of these processes poses a critical challenge from environmental and industrial safety perspectives [1,7].

Spray drying technology has been used for over a century, initially limited to products such as powdered milk and eggs due to its constrained efficiency. However, with ongoing technological advancements, applications for this technique have significantly increased. Currently, it has emerged as a widely employed method across industries including chemical, food, pharmaceutical, biochemical, and material processing [8].

In recent years, spray drying technology has garnered attention for its application in the development of drug delivery systems within the pharmaceutical industry. The spray drying method stands out as one of the foremost techniques for microsphere production. It entails the generation of dry powder by spraying an emulsion or suspension into a stream of hot air within a drying chamber. In this process, the solvent swiftly evaporates, enabling the active ingredient in the suspension to be encapsulated within the material [9,10].

A primary advantage of this method lies in its suitability for highly volatile and thermolabile materials, attributable to the notably brief exposure time to heat. Furthermore, it yields replicable outcomes, boasts low operating costs, and facilitates rapid processing, rendering it an appealing option for encapsulation in diverse industries [11].

The quality of the product is determined by process parameters. These are influenced by key factors such as the inlet temperature, which controls the solvent’s evaporation rate and, consequently, affects both particle formation and the final product’s stability. Another determining factor is the polymer percentage in the formulation, which impact varies depending on the type of polymer used, as it affects the solids content and viscosity. A higher polymer concentration requires more energy for atomization. Additionally, improper feed flow can reduce process efficiency, as particles tend to impact the walls of the drying chamber. Therefore, it is essential to regulate these three factors to optimize the process performance [11,12].

For the development of microspheres, various polymers are employed, each bringing specific characteristics to the microsphere. Certain polymers, such as hydroxypropylmethyl cellulose (HPMC), guar gum (GG), and certain types of inulin (INU), among others, could prolong drug release from the microsphere. Conversely, certain polymers like Eudragit (EU) L100 or S100, as well as hydroxypropylmethyl cellulose phthalate, can delay drug release [12,13]. Others facilitate drug release in specific body regions due to pH effects, such as certain types of Eudragit, or enzymatic effects, like inulin or pectin. However, obtaining microspheres with excellent physical properties is crucial for achieving the optimal results in their development, which can be influenced by polymer selection. Therefore, understanding the impact of different polymers on microsphere formulation is fundamental [14,15].

The following polymers were selected for this study due to their characteristics, biocompatibility, and potential for use in the production of microspheres via spray drying:

Short-chain agave INU: While both natural and modified inulin have been used to create microspheres through various strategies, in this case, short-chain agave inulin was selected. This polymer has not been previously documented in the literature using the spray drying method, providing a unique opportunity to study its behavior under these conditions and gain new insights into its microsphere-forming capacity [12,13].

GG and HPMC: Both polymers are widely used in extended-release formulations and have proven effective in creating microspheres through different methods. They are ideal candidates for evaluating their performance in an aqueous process like spray drying. This will allow us to compare their encapsulation efficiency, release profile, and behavior against inulin, providing a solid basis for their use in controlled-release systems [12,13].

EU S100: This is a pH-dependent polymer that disintegrates in specific media. By using it in an aqueous method where the drug is suspended, we aim to study how this pH-dependent property is expressed during spray drying and whether it affects the drug release in controlled environments [12,13].

In this study, indomethacin is used as a model drug due to its lipophilic nature and the need to improve its therapeutic profile. Formulating indomethacin (IDM) in delayed or sustained-release microspheres aims to reduce the gastric side effects associated with its conventional administration. This is because indomethacin inhibits cyclooxygenase, decreasing prostaglandin production, which plays protective roles in the stomach, such as reducing inflammation and mucus secretion. Therefore, controlled release can minimize gastric damage and improve treatment tolerability.

The objective of this work is to prepare microspheres using spray drying with polymers that have distinct properties, aiming to determine the effect of the process factors and the intrinsic characteristics of the polymers through an experimental design, optimize the process to maximize the encapsulation efficiency and yield, and compare the behavior of the polymers by analyzing their dissolution profiles, thereby establishing relationships between the physicochemical properties of the materials, process parameters, and final performance of the microspheres.

## 2. Results and Discussion

### 2.1. Compatibility Analysis

**Figure 1:**
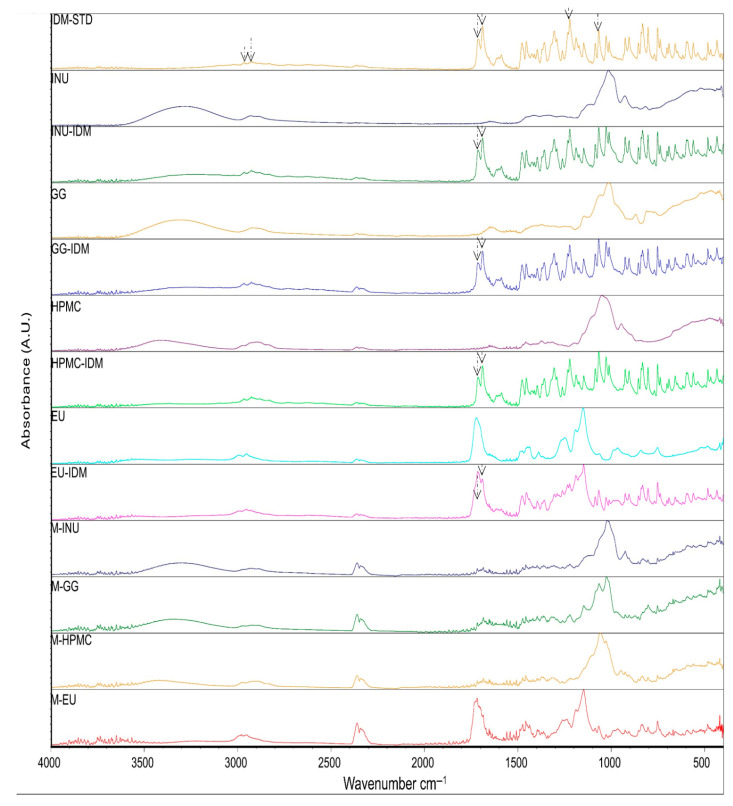
ATR (attenuated total reflection) spectra indomethacin (IDM-STD); polymers: inulin (INU), 1:1 drug–polymer mixture (INU-IDM); guar gum (GG), 1:1 drug–polymer mixture (GG-IDM); hydroxypropyl methylcellulose (HPMC), 1:1 drug–polymer mixture (HPMC-IDM); Eudragit^®^ (EU), 1:1 drug–polymer mixture (EU-IDM) and microspheres: M-INU, M-GG, M-HPM, and M-EU.

The FTIR (Fourier-transform infrared) analysis results showed that the drug IDM exhibits characteristic peaks at specific wavelengths corresponding to different types of chemical bonds in its structure. For instance, signals related to C-H bonds were observed in the regions of 3025 cm^−1^ and 2967 cm^−1^, as well as peaks associated with O-C bonds at 1261 cm^−1^ and 1086 cm^−1^. Additionally, two significant peaks corresponding to carbonyl groups (C=O) were detected: one at 1712.97 cm^−1^, characteristic of the acidic carbonyl group, and another at 1690.39 cm^−1^, related to the amide carbonyl group, attributed to interactions with lone electron pairs of nitrogen. These results indicate that no chemical interaction occurred between the drug IDM and the polymers used, as shown in Figure 1. These signals were reported by Duperyron et al. (2013) [16], who assigned the band at 3025 cm^−1^ to the C-H bonds of the aromatic group and the band at 1712 cm^−1^ to the acidic carbonyl group. Unlike this study, they did not report the signal at 1690.39 cm^−1^ corresponding to the amide carbonyl group [16].

In the IR analysis of the microspheres, the spectra obtained were similar to those of the polymers used in all the formulations, which is consistent with the results reported by Dupeyrón et al., who only detected signals corresponding to EU L100 in their formulations [16]. These authors suggest that the IDM signals are weak because the drug is located inside the microspheres. In our case, we propose that, since the drug is present in a lower proportion compared to the polymer and it is encapsulated within the microspheres, the polymer signals dominate and mask those of the drug. For this reason, an additional technique was applied to confirm the presence of IDM.

**Figure 2:**
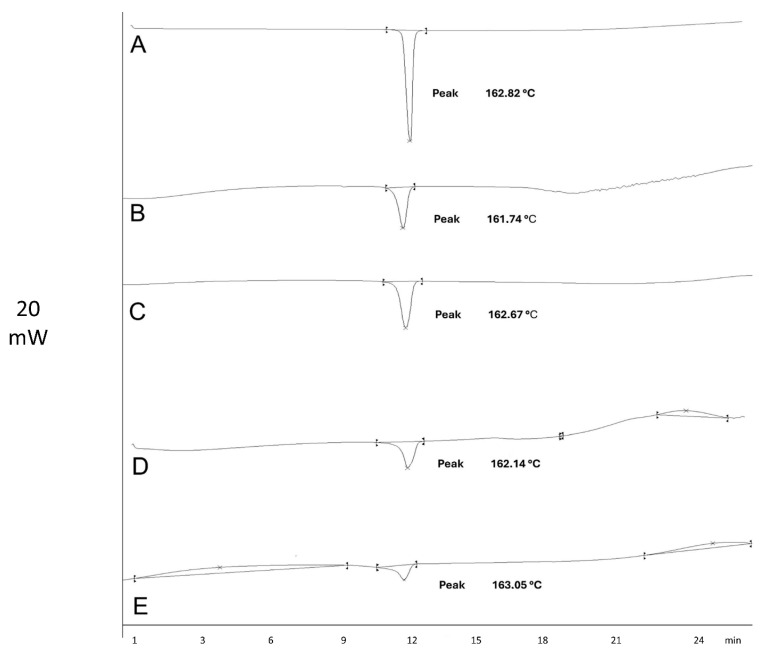
Thermograms of indomethacin (**A**) and microspheres M-INU (**B**), M-EU (**C**), M-HPMC (**D**), and M-GG (**E**).

To verify the inclusion of the drug in the microspheres, differential scanning calorimetry (DSC) analysis was performed. Figure 2 shows the DSC thermograms of the pure drug, polymers, and microspheres. IDM exhibited an endothermic peak at 162.82 °C, corresponding to its melting point. Similar results were obtained by Jain et al. (2014), who reported a peak at 162.26 °C, indicating that the IDM was in its crystalline state [17].

DSC was performed to detect the IDM in the microspheres, identifying the melting point of the drug. The melting point, as reported in the literature, can be used to determine IDM’s polymorphic forms and detect any existing interactions. In this study, the melting point indicated that the IDM used was in its γ polymorphic form [18,19]. In addition, interactions between IDM and the polymers were not identified. IDM remained stable at the operating temperature [17].

### 2.2. Experimental Design Analysis

After analyzing previous research, it was determined that polymers such as HPMC and GG were ideal for working at concentrations between 1% and 1.5% due to their viscosity, which can increase with the suspended drug. Therefore, it was decided to use concentrations below 1% to evaluate other factors without being affected by their high viscosity. In contrast, polymers such as agave INU and EU S100 could be used at concentrations below 10% due to their low viscosity. By including the polymer percentage as a factor to analyze, it was possible to work with different concentrations for each polymer, as the experimental design was carried out individually, facilitating the evaluation of the polymer percentage behavior in each formulation.

Table 1 presents the results of the 2^3^ factorial design for each polymer. The product yield (Y_1_) and encapsulation efficiency (Y_2_) were adjusted to several models using Design-Expert^®^ (DE) software (trial version 11.0.0 Stat-Ease Inc., Minneapolis, MN, USA). The 2FI model (two-factor interaction) was selected based on the *p*-value. Analysis of variance (ANOVA) was applied to estimate the importance of the model, with a 95% confidence level.

Furthermore, GG microspheres demonstrated the highest encapsulation efficiency, averaging 95.9% across the runs, followed by EU at 75.87%, INU at 58.53%, and HPMC at 57.72%. Of these, only guar gum microspheres exhibited statistically significant differences, which will also be discussed in detail in Section 2.3.1.

The results presented in Table 1 indicate that INU microspheres exhibited the highest yield percentage, with an average of 58.53% across the runs, followed by EU microspheres at 38.95%, GG at 25.23%, and HPMC at 23.48%. Among these, only guar gum microspheres showed statistically significant differences, which will be analyzed individually in Section 2.3.2.

It is important to mention that the guar gum microspheres showed the lowest average yield, which is related to the lower polymer percentage used in their formulation. Additionally, this formulation exhibited the most viscous suspension, which likely caused the significant differences observed in the yield. On the other hand, inulin was the polymer used at the highest concentration, with a ratio greater than 2:1 relative to the drug, compared to others that had lower ratios. Furthermore, being the most hydrophilic polymer, it has less affinity for the lipophilic drug, which could explain the significant differences observed in the encapsulation efficiency.

### 2.3. Data Analysis and Optimization

#### 2.3.1. Product Yield

The effects of the factors on product yield (Y_1_) in the GG formulation were evaluated using ANOVA with the adjusted model Y= log_10_ (Y + k) with k = 0; 2FI modified: Y = b_1_X_1_ + b_2_X_2_ + b_3_X_3_ + b_12_X_1_X_2_ + b_13_X_1_X_3_ revealed significant differences (Table 2).

**Figure 3:**
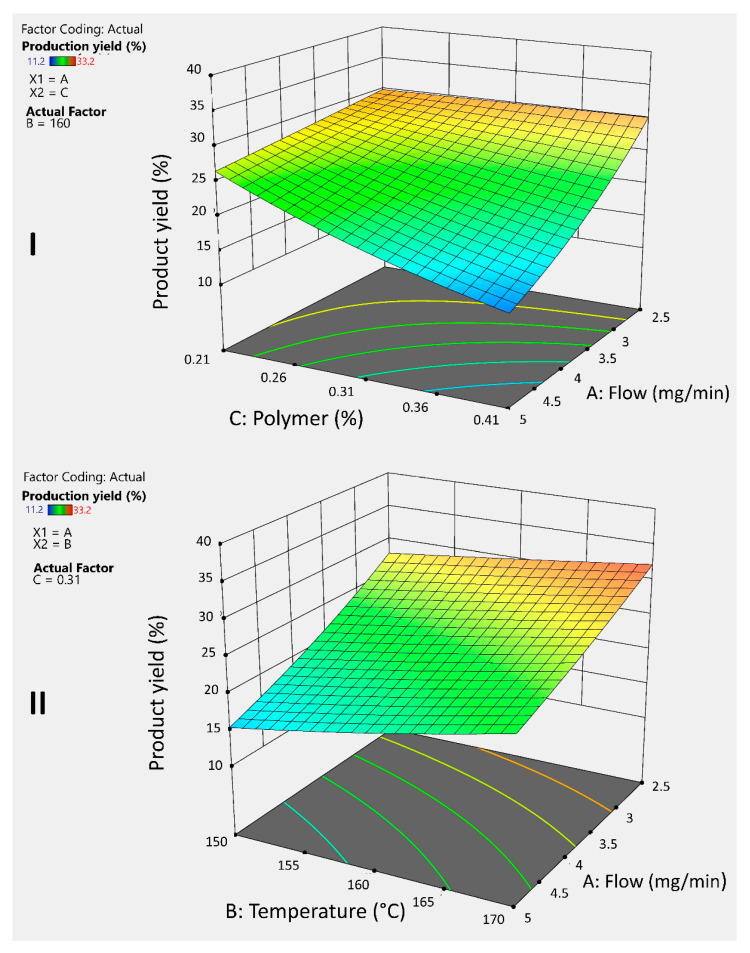
3D graphs illustrates the response and interaction of the AC factors (**I**) and AB factors (**II**) on the response variable Y_1_ for guar gum microspheres.

Figure 3I shows that both the polymer percentage and flow rate have a negative effect on the yield, as increasing these factors leads to a decrease in the response. Furthermore, the simultaneous increase of both factors produces a negative synergistic interaction, further reducing the yield. This behavior is consistent with previous reports in the literature, since a higher polymer percentage in the formulation increases the viscosity, causing part of the atomized liquid to adhere to the walls of the drying chamber. A similar effect is observed when the flow rate is increased. Shah and colleagues (2015) shared this statement, as they observed the same behavior when producing microspheres with chitosan by spray drying [20,21].

To counteract this effect, it is essential to evaporate the solvent as quickly as possible and prevent its adhesion to the drying chamber; temperature plays a crucial role in this process. Figure 3II shows that higher temperatures improve the yield, offsetting the negative effect of the flow rate. Moreover, combining a reduced flow rate with an elevated temperature results in a positive interaction that allows achieving the highest yield.

#### 2.3.2. Encapsulation Efficiency

The effects of the factors on the encapsulation efficiency (Y_2_) in the INU formulation were evaluated using ANOVA with the adjusted model Y = log_10_ (Y + k) with k = 0; 2FI modified: Y = b_1_X_1_ + b_2_X_2_ + b_3_X_3_ + b_12_X_1_X_2_ + b_13_X_1_X_3_ + b_23_X_2_X_3_ revealed significant differences (Table 3).

**Figure 4:**
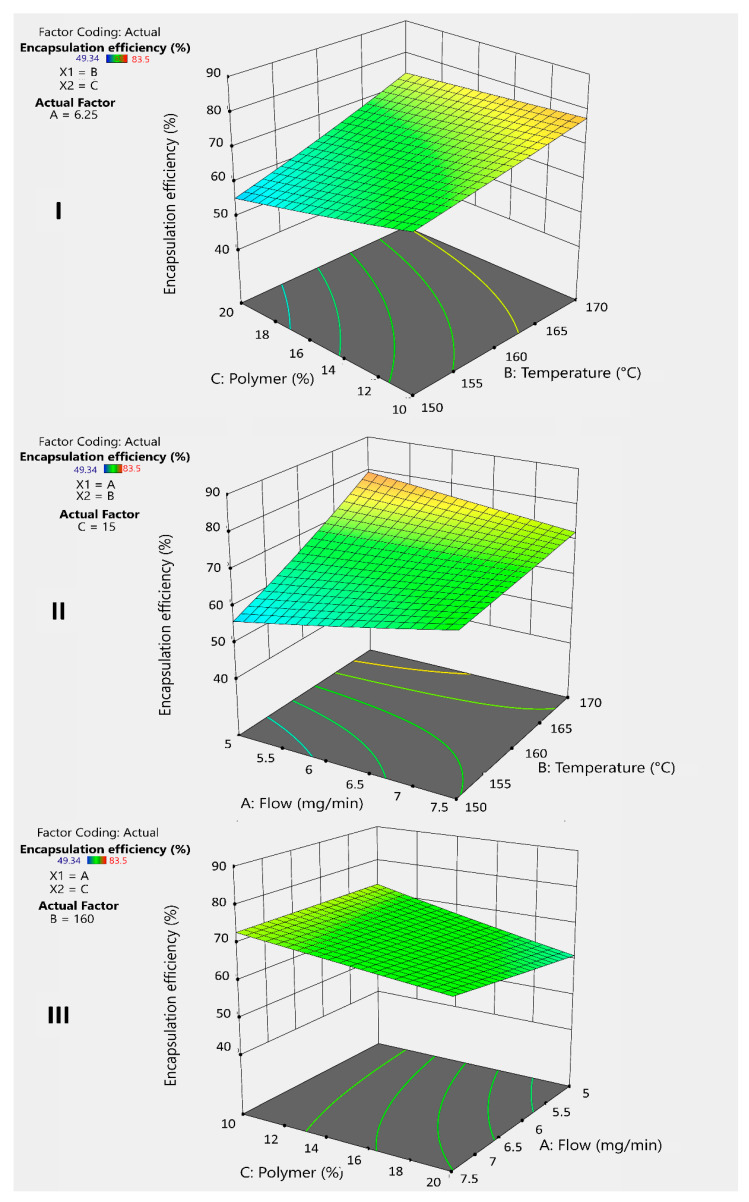
3D graphs illustrates the response and interaction of factors BC (**I**), BA (**II**), and AC (**III**) on the response variable Y_2_ for inulin microspheres.

Figure 4I shows that the polymer percentage has a negative effect on the encapsulation efficiency. This is due to the low affinity between inulin (INU) and the drug, as inulin is hydrophilic, while the drug is lipophilic. Dupeyrón et al. (2013) [16] suggested that the high encapsulation efficiency observed in their study was due to the affinity between the drug and the polymer when both are lipophilic, as they tend to avoid the external aqueous phase. Analyzing the average results from the experiments, more lipophilic polymers, such as Eudragit^®^ (EU) and guar gum (GG), exhibited better performance in this parameter [16].

To counteract this effect, temperature plays an important positive role (Figure 4I,II) by accelerating solvent evaporation and facilitating microsphere formation. The faster this process occurs, the greater the drug retention within the microsphere, compensating for the lack of affinity. Additionally, combining a lower polymer percentage with an increased temperature generates a positive interaction that improves the encapsulation efficiency (Figure 4I).

On the other hand, the flow rate has a minimal but positive effect (Figure 4II), sufficient to accelerate microsphere formation when combined with increased temperature. This combination produces a positive synergistic interaction that helps improve drug encapsulation (Figure 4II). Similarly, increasing the flow rate while decreasing the polymer percentage results in a better encapsulation efficiency (Figure 4III). However, this strategy may negatively affect the yield.

After the statistical analysis, the DE software recommended the following optimal conditions: an inlet temperature of 170 °C, as it showed a positive effect on both response variables, and a feed rate of 5 mg/min. Table 4 presents the formulations with the optimal polymer percentage, which, in this case, corresponds to the low value. These settings maximize the encapsulation efficiency without significantly compromising the process yield. The results obtained are analyzed in Section 2.4.

### 2.4. Comparative Analysis of the Polymers

**Figure 5:**
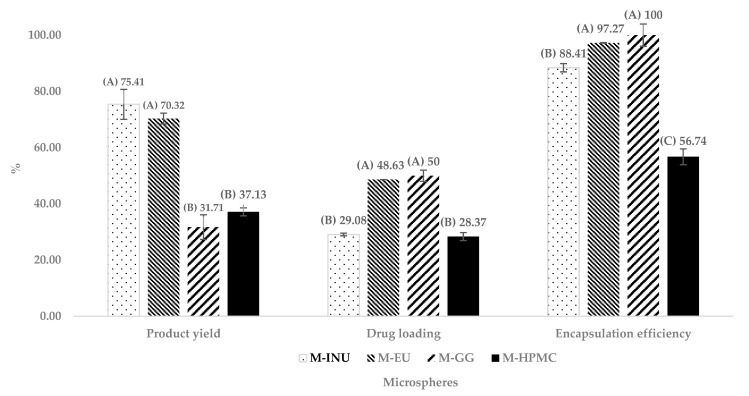
Comparison of the product yield, drug loading, and encapsulation efficiency of optimized microspheres. Microspheres of inulin (M-INU), Eudragit^®^ S100 (M-EU), guar gum (M-GG), and hydroxypropyl methylcellulose (M-HPMC) (n = 3). Two statistically different groups were identified in the product yield and drug loading and three in the encapsulation efficiency, and those in group (A) presented better results, followed by those in group (B) and (C) (*p* ˂ 0.05).

Figure 5 summarizes the performance of the optimized formulations in Section 2.3.2, the results improved compared to the average of the runs in the experimental design.

M-INU achieved the highest yield (Y_1_) (75.40%), followed by M-EU (70.30%). In contrast, M-HPMC and M-GG recovered only 37.10% and 31.70%, respectively. According to Tukey’s test (*p* < 0.05), these differences were statistically significant, forming two groups: one with the higher-performing formulations, M-INU and M-EU, which were not statistically different from each other, and another group with lower results, consisting of M-GG and M-HPMC. The higher yield of inulin and Eudragit^®^ is attributed to their low viscosity, which minimizes wall deposition during spray drying [18,20].

Regarding drug loading, M-GG showed the highest values, closely followed by M-EU, with percentages of 50% and 48%, respectively. These were followed by M-INU and M-HPMC with 29.08% and 28.37%, respectively. Tukey’s test revealed statistically significant differences, forming two groups: one with the higher-performing formulations, M-EU and M-GG, which did not differ significantly from each other, and another group with the lower values, consisting of M-INU and M-HPMC.

On the other hand, for the encapsulation efficiency (Y_2_), three statistically different groups were identified: the first, with the best results, included M-GG (100%) and M-EU (97.27%); the second group consisted of M-INU (88.41%); and the third group, with the lowest performance, corresponded to M-HPMC (56.74%).

The excellent performance of GG and EU is attributed to their hydrophobic domains, which exhibit a higher affinity for indomethacin (log P ≈ 4.3).

INU and EU deliver high yields, because their low viscosity allows complete atomization and prevents sticking. Conversely, GG and HPMC suffer greater losses. Regarding encapsulation, hydrophobic affinity dominates: GG > EU > INU > HPMC [17]. The poorer performance of HPMC is further aggravated by its irregular, collapsed particles, which reduce the effective drug-trapping volume [16].

Collectively, these results indicate that low viscosity favors the yield, whereas hydrophobic affinity favors encapsulation, with EU providing the best trade-off between the two requirements.

### 2.5. Morphology

**Figure 6:**
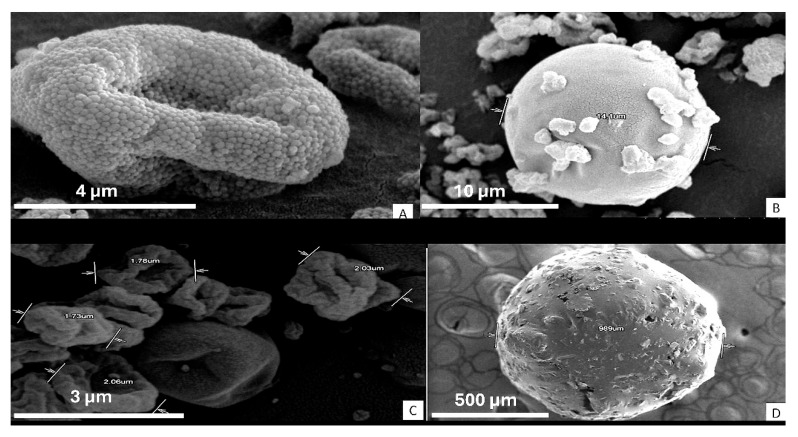
SEM microspheres: Eudragit S100 (**A**), guar gum (**B**), hydroxypropyl methylcellulose (**C**), and inulin (**D**).

Figure 6 presents scanning electron microscope (SEM) micrographs of the microspheres obtained under the optimized parameters. The final morphology depends on the polymer type and the evaporation dynamics during spray drying:

M-EU exhibits a granular and rough surface (Figure 6A), attributable to the polymer’s partial solubility in water, which leads to poorly fused solid domains, negatively affecting its ability to sustain drug release [16,21].

M-GG forms spherical and smooth microspheres with small drug particles on the surface, consistent with reports by Kuck and Noreña for partially hydrolyzed guar gum (Figure 6B) [22,23].

M-HPMC shows irregular particles of 2–3 µm with an apparently smooth but “wrinkled” surface (Figure 6C). Previous studies indicate that, at elevated temperatures (such as the 170 °C used in this study), the solvent trapped within the particle crust is abruptly released, causing localized collapses [21,24].

M-INU forms an agglomerate larger than 900 µm due to the polymer’s high hygroscopicity, which promotes post-drying adhesion (Figure 6D). Other authors have demonstrated that the methylation of inulin reduces this tendency and allows the formation of discrete spheres (~4 µm) [25,26].

These observations confirm that (i) low polymer solubility or high viscosity result in rough or collapsed surfaces, and (ii) high hygroscopicity induces agglomeration after drying and spherical morphology promotes encapsulation. Quantitative characterization (mean diameter and circularity) and its correlation with the yield and release profile will be addressed in future studies.

### 2.6. Dissolution Kinetics

**Figure 7:**
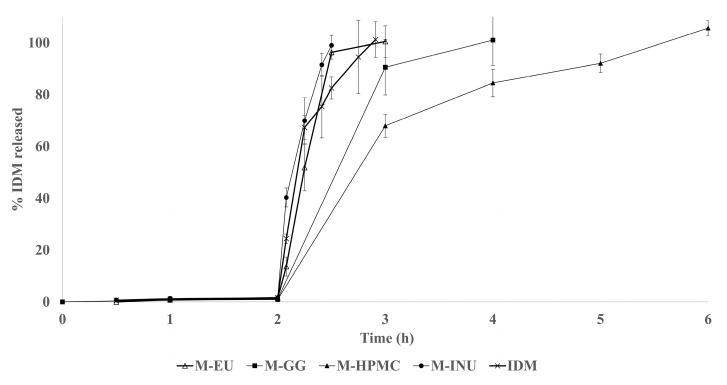
Profile released of indomethacin (IDM) of the microspheres: inulin (M-INU), Eudragit^®^ S100 (M-EU), guar gum (M-GG), and hydroxypropyl methylcellulose (M-HPMC).

Figure 7 compares the in vitro dissolution profiles of each formulation following validation of the UV–Vis assay (r^2^ = 0.999, CV = 1.22%).

For the dissolution profile, the microspheres from each formulation were weighed. The amount required to contain 150 mg of drug was calculated based on the drug loading values, resulting in 516 mg for M-INU, 309 mg for M-EU, 300 mg for M-GG, and 530 mg for M-HPMC.

IDM was not detected during the acidic stage (pH 1.2) due to its practically insoluble nature in this medium. Upon shifting to pH 6.8, the unformulated drug achieved complete dissolution within 55 min. In contrast, M-INU released 100% of the payload within 30 min, indicating that short-chain agave INU acts as a humectant, accelerating drug dissolution. This behavior markedly differs from that reported by Poulain et al. (2003) [26], where dahlia INU microspheres released only 65% of the drug in the first 5 min (pH 7.4), with the remaining 35% released over more than three days. This discrepancy is attributed to structural differences: dahlia inulin is more linear and less branched, forming denser gels that slow the release [24,27].

Guar gum (GG) and HPMC microspheres exhibited a rapid drug release known as the “burst” effect due to the drug distributed on the microsphere surface, as observed by scanning electron microscopy (SEM), and the drug trapped near the surface. Subsequently, the release was prolonged as the drug diffused from the core. However, the formation of the polymer’s three-dimensional gel was not sufficiently strong due to its low concentration in the formulation, resulting in 80% release during the first hour of the second stage (pH 6.8), reaching 100% at 4 and 6 h for GG and HPMC, respectively. The release times obtained in this study are shorter than those reported by Kumar et al., who achieved complete release in approximately 10 h using GG and HPMC microspheres formulated at a higher polymer concentration. It is important to note that Kumar and colleagues employed a solvent evaporation method with organic solvents, whereas our work used an aqueous method. These differences in technique and polymer concentration may explain the variations observed in the release profiles [28].

These matrix system characteristics caused simultaneous diffusion and erosion release, known as anomalous transport (n > 1) (Table 5). Inulin (INU) can be used for immediate release, while HPMC and GG require a higher polymer percentage in the formulation to achieve prolonged release, although this would negatively affect the production yield. Alternatively, encapsulation or tablet formulation is suggested [24,27].

Although EU S100 is designed to dissolve above pH 7.0, the M-EU produced released the drug rapidly, achieving 100% dissolution within 60 min at pH 6.8 [29]. SEM images revealed surface micropores, likely generated by incomplete polymer solubilization during aqueous processing; these insoluble particle junctions probably facilitate water ingress, early particle disintegration, and accelerated drug release. In contrast, Deore et al. (2013) prepared smooth, spherical EU S100/L100 (1:1) microspheres via an emulsion–solvent evaporation method, achieving a significantly slower release profile, underscoring the critical impact of the manufacturing method on performance [30].

## 3. Materials and Methods

### 3.1. Materials

IDM ≥98% purity pharmaceutical grade, used as lipophilic model drug, was donated from Laboratory Arlex (Mexico City, Mexico). Agave INU (88–94%) of Alit Powder^®^ fiber brand was supplied by Alit^®^ (Guadalajara, Mexico). GG (powder) was acquired in Development of Chemical Specialties (Monterrey, Mexico). HPMC SHEFFCEL^®^ 75 HD15000 (4000 cps, K4M) of KERRY brand was obtained through Chemcel, S.A. de C.V. (Mexico City, Mexico). EU S100 was provided by Helm de México S.A. (Estado de Mexico, Mexico), while other commercially available reagents of analytical grade were obtained for this study.

### 3.2. Microspheres Preparation

**Figure 8:**
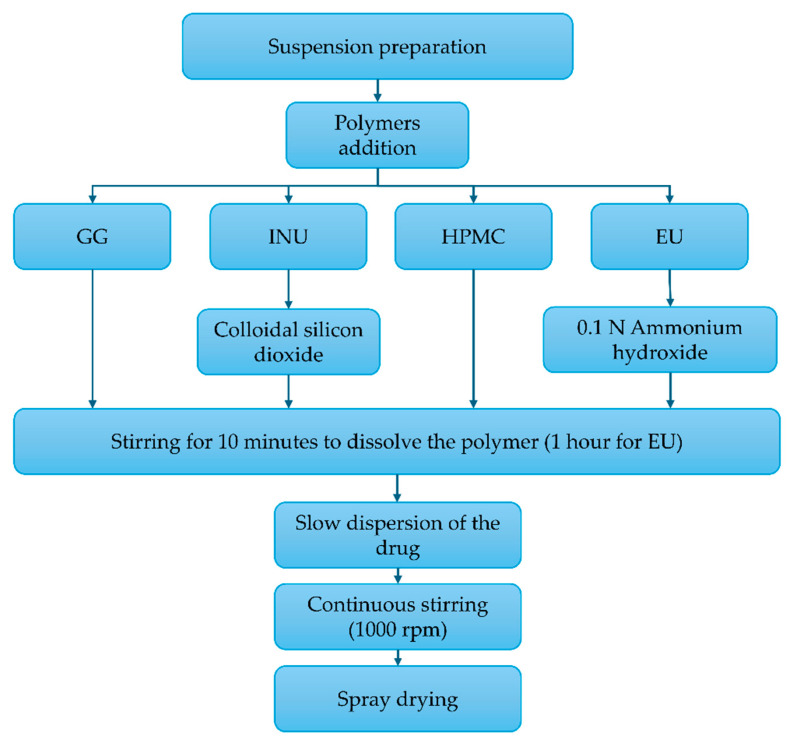
Process for the preparation of the microspheres: inulin (INU), Eudragit^®^ S100 (EU), guar gum (GG), and hydroxypropyl methylcellulose (HPMC).

Microspheres were prepared according to the formulations shown in Table 6, following the procedure described in Figure 8, using the Büchi B290 spray dryer (BUCHI, Mexico City, Mexico). The operating parameters were customized for each polymer, following an experimental 2^3^ design. The aspiration rate (100%) and inlet air pressure (742 L/h) were maintained as constant throughout each process [31].

### 3.3. Compatibility Determination

The pure drug, polymers, binary mixtures 1:1 IDM:polymer, and microspheres were subjected to compatibility determination by attenuated total reflection Fourier-transform infrared spectroscopy (PerkinElmer^®^ Universal ATR Sample Accessory Spectrum ONE FTIR Spectrometer; PerkinElmer, Waltham, MA, USA). Additionally, a complementary DSC was performed as follows: 2 mg of each sample were weighed, placed in aluminum capsules sealed, and transferred to a Shimadzu DSC50 (Shimadzu Scientific instruments, Kioto, Japon) differential scanning calorimeter at a heating rate of 10 °C/min over a temperature range of 50–300 °C with a constant nitrogen flow of 30 mL/min [32].

### 3.4. Experimental Design

A 2^3^ factorial design was employed, considering three factors with two levels each (high and low), resulting in eight combinations for each formulation (Table 7) (Design-Expert^®^ (DE) software (trial version 11.0.0 Stat-Ease Inc., Minneapolis, MN, USA)). The factors evaluated were feed flow (X1), inlet temperature (X2), and polymer percentage (X3). The measured responses were the product yield (Y_1_) and encapsulation efficiency (Y_2_), as these directly impact the manufacturing process. These variables help to assess the utilization of raw materials and quantify the amount of drug present in the microspheres, which is crucial for accurate dosing.

The experimental design used IDM as a model drug for all the microsphere-forming polymers (INU, GG, HPMC, and EU). All other formulations and processing variables remained constant throughout the study.

### 3.5. Data Analysis and Optimization

ANOVA was used to statistically validate the polynomial equations generated by DE software. Additionally, a multiple linear regression model based on a 2^3^ factorial design was applied to obtain a prediction equation to evaluate the responses (Equation (1)):

(1) $$Y=b_{0}+b_{1}X_{1}+b_{2}X_{2}+b_{3}X_{3}+b_{12}X_{1}X_{2}+b_{13}X_{1}X_{3}+b_{23}X_{2}X_{3}$$

where:

- Y is the response measured according to the combination of factor levels;
- b_0_ is the average value of all measurements;
- b_1_, b_2_, and b_3_ are coefficients representing the individual effects of each factor;
- b_12_, b_13_, and b_23_ are coefficients representing the interactions between pairs of factors;
- X_1_, X_2_, and X_3_ are the coded levels of the independent factors.

The main effects (X_1_, X_2_, and X_3_) show the response changes when a single factor varies from its low to high level. Interaction terms indicate how the response changes when two factors vary simultaneously [20].

This model was used to analyze the product yield and encapsulation efficiency responses for each formulation, selecting the best fit based on statistical parameters including the coefficient of variation, the multiple correlation coefficient (R^2^), the adjusted multiple correlation coefficient (adjusted R^2^), and the predicted residual sum of squares, with a confidence level of *p* < 0.05. Main effect plots were generated, and a desirability approach was applied to determine the optimal conditions for the polymer formulations.

#### Comparative Analysis of the Polymers

A comparative analysis of the results of the microspheres obtained from the optimized process was performed using Tukey’s test to determine the significant differences (*p* < 0.05) between the INU, GG, HPMC, and EU polymers.

### 3.6. Microsperes Characterization

#### 3.6.1. Product Yield

The first step was to weigh the polymers and IDM before preparing the suspension and then the total drug-loaded microspheres. The product yield was calculated with the following equation (Equation (2)) [33]:

Product yield (%Y) = Initial Weight/Weight of total prepared microspheres × 100(2)

#### 3.6.2. Encapsulation Efficiency and Drug Loading

A modification of the methodology applied by Gangane and Kawtikwar 2020 [34] was employed as a percentage encapsulation efficiency technique. A sample of 20 mg of microspheres equivalent to 10 mg of the drug was dissolved in 10 mL of methanol in a 100-mL volumetric flask; the final volume was adjusted with phosphate buffer, pH 7.4. The solution was filtered and analyzed spectrophotometrically at 320 nanometers (nm) (Varian Inc. Carry 50, Cary, NC, USA), and the method was previously validated. The encapsulation efficiency and drug loading were calculated with the following equations (Equations (3) and (4)):

Encapsulation efficiency (EE) = Actual amount of drug encapsulated/Theorical amount of drug in the microspheres × 100(3)

Drug loading (DL) = Drug content in microspheres/Weight of total prepared microspheres × 100(4)

#### 3.6.3. SEM Morphology

SEM analysis was performed to observe the surface morphology of the microspheres. Each sample was screened by passing the material through 200 mesh grids attached to adhesive carbon tape and fixed to aluminum SEM stubs. In addition, each sample was coated with a 30-nm-thick gold layer using an Eiko IB-5 ion coater (EIKO, Ibaraki, Japan) to increase the material’s conductivity. The SEM micrographs were acquired using a Hitachi 3700N electron microscope (Hitachi, Tokyo, Japan) at 15 Kv in a high-vacuum system [34].

#### 3.6.4. Dissolution Profile and Kinetics

The in vitro dissolution experiments were performed using drug dissolution USP Apparatus II at 100 rpm and 37 °C. At the beginning of the test, a specific weight of microspheres equivalent to 150 mg of the drug was directly added to the glass vessel. The dissolution process had three stages: the first over 2 h in 0.1 N chlorohydric acid (750 mL) medium, the second over 3 h in an alkalinized medium (pH 6.8) with 0.2 M sodium phosphate tribasic solution, and the third over 5 h in a medium adjusted to pH 7.4 with 2 N sodium hydroxide. The experiment was done in triplicate. Samples were taken hourly from the central area of the medium and filtered through a 33-mm diameter 0.45-µm pore size polyvinylidene fluoride membrane filter (Millipore, Burlington, MA, USA). Next, the amount of IDM released was measured using ultraviolet–visible (UV–Vis) spectrophotometry at 320 nm (Varian Inc. Carry 50) with a previously validated method. Finally, the percentage of IDM released at each sampling stage was calculated [35,36]. The release kinetics were analyzed to determine the predominant release mechanism.

## 4. Conclusions

In this study, indomethacin microspheres were prepared using four different polymers: inulin, guar gum, HPMC, and Eudragit S100. Spectroscopic analysis and differential scanning calorimetry confirmed the absence of chemical interactions between the drug and the polymers, indicating that indomethacin remained stable within the formulations.

The 2^3^ factorial experimental design revealed that the encapsulation efficiency was significantly affected only in the inulin microspheres, where temperature positively influenced the encapsulation, while the drug percentage and flow rate had negative effects. Regarding yield, only the guar gum microspheres showed significant differences, with temperature acting positively and flow rate and polymer percentage negatively, likely due to the polymer’s high viscosity.

Particle size and morphology varied depending on the polymer: inulin produced larger particles, whereas HPMC generated smaller, irregularly shaped microspheres, correlating with a lower encapsulation efficiency. The guar gum microspheres were more spherical, which likely contributed to better drug encapsulation, possibly due to the lipophilic affinity with indomethacin.

Regarding the drug release profile, rapid release was observed in all cases, with approximately 80% of the drug released within the first hour at pH 6.8. Inulin released the drug almost immediately, while Eudragit did not exhibit the expected delayed release, probably due to the limitations of the aqueous manufacturing method. Guar gum and HPMC extended the release by only a few additional hours, insufficient to be considered a truly sustained release under the tested conditions.

These findings indicate that, although the selected polymers and method allowed the formation of stable microspheres with good encapsulation, achieving controlled-release profiles requires further optimization, such as increasing the polymer concentration or modifying the manufacturing process. This work provides an experimental foundation for future improvements, including evaluating different solvents, polymer combinations, and conducting stability and in vivo efficacy studies, which are necessary to develop more effective delivery systems for lipophilic drugs like indomethacin.

## Figures and Tables

**Table 1:** Results of the product yield (%Y) and encapsulation efficiency (%EE) of all the formulations.

Test	Microspheres							
	Inulin		Eudragit^®^ S100		Guar Gum		HPMC	
	% Y	% EE	% Y	% EE	% Y	% EE	% Y	% EE
R1	60.83	63.69	37.60	69.38	28.00	100.59	24.40	55.29
R2	60.40	73.37	20.00	87.52	21.20	100.77	24.00	64.41
R3	52.76	83.50	43.20	82.31	32.00	101.63	24.00	67.43
R4	61.97	72.42	34.00	78.06	33.20	92.30	28.00	68.38
R5	62.38	49.34	46.66	71.09	28.00	99.63	22.93	57.87
R6	45.24	62.37	41.33	66.80	11.20	101.69	22.93	58.37
R7	63.57	75.8	55.47	70.67	32.53	83.92	20.53	57.65
R8	61.11	72.22	33.33	81.17	15.73	86.93	21.06	32.43
*p*-value model (*p* < 0.05)	0.2416	0.0017	0.5933	0.9315	0.0069	0.4711	0.3694	0.4500

**Table 2:** ANOVA of the factorial model of Y_1_.

Source	Sum of Squares	df	Mean Square	F-Value	*p*-Value	
Model	0.2073	5	0.0415	144.98	0.0069	significant
A-Flow	0.0837	1	0.0837	292.76	0.0034	
B-Temperature	0.0271	1	0.0271	94.70	0.0104	
C-Polymer	0.0442	1	0.0442	154.46	0.0064	
AB	0.0060	1	0.0060	21.00	0.0445	
AC	0.0463	1	0.0463	161.96	0.0061	
Residual	0.0006	2	0.0003			
Cor Total	0.2078	7				

R^2^ = 0.9972, adjusted R^2^ = 0.9904, and predicted R^2^ = 0.9560.

**Table 3:** ANOVA of the factorial model of Y_2_.

Source	Sum of Squares	Df	Mean Square	F-Value	*p*-Value	
Model	0.0343	6	0.0057	1.928 × 10^5^	0.0017	significant
A-Flow	0.0008	1	0.0008	27,255.45	0.0039	
B-Temperature	0.0164	1	0.0164	5.531 × 10^5^	0.0009	
C-Polymer	0.0063	1	0.0063	2.128 × 10^5^	0.0014	
AB	0.0076	1	0.0076	2.554 × 10^5^	0.0013	
AC	0.0008	1	0.0008	27,774.20	0.0038	
BC	0.0024	1	0.0024	80,557.60	0.0022	
Residual	2.963 × 10^−8^	1	2.963 × 10^−8^			
Cor Total	0.0343	7				

R^2^ = 0.9999, adjusted R^2^ = 0.9999, and predicted R^2^ = 0.9999.

**Table 4:** Optimal formulations proposed by the experimental design.

M-INU		
Components	Amount (g)	Percentage (%)
Inulin	10	10
Indomethacin	5	5
Colloidal silicon dioxide	0.2	0.2
Water	84.8	84.8
Total	100	100
**M-GG**		
**Components**	**Amount (g)**	**Percentage (%)**
Guar gum	1.25	0.21
Indomethacin	1.25	0.21
Water	597.5	99.58
Total	600	100
**M-HPMC**		
**Components**	**Amount (g)**	**Percentage (%)**
HPMC	1.25	0.31
Indomethacin	1.25	0.31
Water	397.50	99.38
Total	400	100
**M-EU**		
**Components**	**Amount (g)**	**Percentage (%)**
Eudragit^®^ S100	2.5	2.5
Indomethacin	2.5	2.5
Ammonium hydroxide 1N	1.25	1.25
Water	93.75	93.75
Total	100	100

**Table 5:** Release kinetics of M-GG (microspheres guar gum) and M-HPMC (microspheres hydroxypropyl methylcellulose).

Formulation	Zero Order	First Order	Higuchi	Korsmeyer–Peppas	
	R^2^	R^2^	R^2^	R^2^	n
M-GG	0.8418	0.8895	0.8142	0.8147	4.17
M-HPMC	0.8091	0.9622	0.8625	0.7161	4.01

**Table 6:** Formulation of the microspheres.

M-INU		
Components	Amount (g)	Percentage (%)
Inulin	10/20	10/20
Indomethacin	5	5
Colloidal silicon dioxide	0.2	0.2
Water	84.8/74.8	84.8/74.8
Total	100	100
**M-GG**		
**Components**	**Amount (g)**	**Percentage (%)**
Guar gum	1.25/2.5	0.21/0.42
Indomethacin	1.25	0.21
Water	597.5/596.5	99.58/99.37
Total	600	100
**M-HPMC**		
**Components**	**Amount (g)**	**Percentage (%)**
HPMC	1.25/2.5	0.31/0.63
Indomethacin	1.25	0.31
Water	397.50/396.25	99.38/99.06
Total	400	100
**M-EU**		
**Components**	**Amount (g)**	**Percentage (%)**
Eudragit^®^ S100	2.5/5	2.5/5
Indomethacin	2.5	2.5
Ammonium hydroxide 1N	1.25/2.5	1.25/2.5
Water	93.75/90	93.75/90
Total	100	100

**Table 7:** Combination of the factors and their levels in the formulations. Inulin microspheres (M-INU), guar gum microspheres (M-GG), HPMC microspheres (M-HPMC), and Eudragit^®^ S100 microspheres (M-EU).

Test	M-INU			M-GG			M-HPMC			M-EU		
	Factors Level											
	X_1_	X_2_	X_3_	X_1_	X_2_	X_3_	X_1_	X_2_	X_3_	X_1_	X_2_	X_3_
**R1**	5	150	10	2.5	150	0.21	5	150	0.31	2.5	150	2.5
**R2**	7.5	150	10	5	150	0.21	7.5	150	0.31	5	150	2.5
**R3**	5	170	10	2.5	170	0.21	5	170	0.31	2.5	170	2.5
**R4**	7.5	170	10	5	170	0.21	7.5	170	0.31	5	170	2.5
**R5**	5	150	20	2.5	150	0.42	5	150	0.63	2.5	150	5
**R6**	7.5	150	20	5	150	0.42	7.5	150	0.63	5	150	5
**R7**	5	170	20	2.5	170	0.42	5	170	0.63	2.5	170	5
**R8**	7.5	170	20	5	170	0.42	7.5	170	0.63	5	170	5

Factors: X_1_: feed flow (mg/min); X_2_: inlet temperature (°C); X_3_: polymer (%).

## Data Availability

Data is contained in the paper.

## Abbreviations

The following abbreviations are used in this manuscript:

IDM	indomethacin
INU	inulin
GG	guar gum
HPMC	hydroxypropyl methylcellulose
EU	Eudragit
FTIR	Fourier-transform infrared
DSC	differential scanning calorimetry
ANOVA	analysis of variance
DE	Design-Expert^®^
ATR	attenuated total reflection
SEM	scanning electron microscope
